# Gestures in Storytelling by Preschool Chinese-Speaking Children With and Without Autism

**DOI:** 10.3389/fpsyg.2020.573212

**Published:** 2020-09-08

**Authors:** Ying Huang, Miranda Kit-Yi Wong, Wan-Yi Lam, Chun-Ho Cheng, Wing-Chee So

**Affiliations:** Department of Educational Psychology, The Chinese University of Hong Kong, Hong Kong, China

**Keywords:** gesture, autism spectrum disorder, storytelling, emblem, supplementary relation

## Abstract

Previous findings on gestural impairment in autism are inconsistent, while scant evidence came from Chinese-speaking individuals. In the present study, preschool Chinese-speaking children with typical development and with autism were asked to generate stories from a set of wordless Cartoon pictures. Two groups were matched in chronological age and language developmental age. Their speech and gestures were coded. Compared to children with typical development, children with autism produced fewer gestures and showed lower gesture rate. Besides, children with autism produced fewer emblems and fewer supplementary gestures compared to their TD peers. Unlike children with typical development, children with autism tend to produce emblems for reinforcing, rather than supplementing information not conveyed in speech. Results showed the impairments in integrating the cross-modal semantic information in children with autism.

## Introduction

Children typically exhibit communicative behaviors during their first year. Although spoken words become a preferred form of communication after the first year of development, children continue to gesture to reinforce or extend spoken messages or even to replace them (Colletta and Guidetti, 2012). Gestures refer to actions that are made with the intention of communication, and they can involve the hands, the fingers, and the whole body (Bochner and Jones, 2003). Gestural skills are crucial for facilitating communication. Gestures provide semantic information in a visual format (Goldin-Meadow, 2006) and help listeners understand speech better, especially when the co-occurring speech underspecifies information (Hostetter, 2011).

In comparison to children without autism spectrum disorder (ASD), children with ASD have a delay in verbal and nonverbal communication skills (Lai et al., 2014). Most of the children diagnosed with autism disorder show a significant delay in language development (Tager-Flusberg et al., 2005). Impairments in nonverbal skills, such as the use of gestures, from early childhood to school age in children with ASD have also been reported. In comparison to their typically developing (TD) peers, children with ASD have deficits in understanding and producing gestures (Colgan et al., 2006; Mitchell et al., 2006). Preschool children with ASD rarely use deictic gestures (i.e., pointing) to attract others’ attention or to share their interest with others (Camaioni et al., 2003). Compared to mentally retarded children matched on mental age or language age, children with ASD showed deficits in gestural joint attention skills, which predicted their language development (Mundy et al., 1990). They have difficulties in understanding and producing conventional gestures (also known as emblems), such as waving hands to represent “goodbye” (Stone et al., 1997; Wetherby et al., 1998). In addition, the production of iconic and beat gestures is delayed in children with ASD (Charman et al., 2003; Wetherby et al., 2004; Luyster et al., 2007). It is also found that children with ASD imitate gestures worse than TD children and are more likely to make errors (Smith, 1998). Moreover, school-aged children with ASD are less able to perceive and produce gestures (Schreibman et al., 2015; So et al., 2015). It was reported that adolescents with ASD produce fewer metaphorical and beat gestures than their TD peers with matched age and verbal IQ (Morett et al., 2016). Researchers have argued that although verbally fluent teenagers use the same type of gestures as their TD peers, their gestures are more difficult to understand (Silverman et al., 2017).

However, evidence of impairment in the use of gestures is inconsistent across studies. For example, Mastrogiuseppe et al. (2015) reported that the amount of gestures produced by children with ASD is significantly lower than TD children and children with Down Syndrome. Conversely, Wong and So (2018) found that, compared to TD children, children with ASD produce a similar number of pointing gestures and markers and more iconic gestures. Similarly, de Marchena et al. (2019) reported that adults with autism used gestures more than TD controls for regulating conversational dynamics. When examining gesture rates (number of gestures per utterance), some researchers reported lower gestures rates in the ASD group (So et al., 2015; Morett et al., 2016; Silverman et al., 2017) while others found comparable gesture rates between the ASD group and the TD group (de Marchena and Eigsti, 2010). Similarly, findings on gesture types also vary. So et al. (2015) found that children with ASD use fewer types of gestures, while Silverman et al. (2017) suggest that the proportion of gesture types did not differ between the ASD group and TD group. In regard to gesture quality and meaning, Morett et al. (2016) and So et al. (2015) reported fewer, or even the absence of, supplementary gestures in children with ASD. However, Wong and So (2018) found that children with ASD produced comparable supplementary gestures to TD children. Moreover, the use of gestures may vary across cultures (Kita, 2009).

Most of the previous studies on gestural skills in individuals with ASD are based on English-speaking participants. Some recent studies reporting delayed and deficit in gestural use in school-aged Chinese-speaking participants with ASD (So et al., 2015, 2016, 2018). These results suggesting that early intervention is critical. However, little is known about the use of gestures and gestural skills in preschool Chinese-speaking children with or without ASD. This study examined whether Chinese-speaking children with ASD had impairments in gestural production skills compared to their age-matched TD peers. A narrative elicitation task was used to assess the rates, types, and gesture-speech relation produced spontaneously during storytelling. We expected that results would be consistent with previous findings of So et al. (2015). Specifically, we expected that children with ASD would produce fewer gestures, especially fewer emblems, and fewer supplementary gestures than their age-matched TD peers. Results could provide evidence for designing effective intervention programs.

## Methods

### Participants

Twenty children with ASD and 14 TD children participated in the current study. All participants were native speakers of Chinese (Cantonese) aged 4 to 6. Children in the ASD group had been diagnosed with autism or autistic disorder by pediatricians at the Child Assessment Center for the Department of Health in Hong Kong. All procedures in the present study were approved by the institutional review board of the author’s university, in compliance with the Declaration of Helsinki (Reference no. 14600817). Before the study, we explained the procedures to the parents and obtained their approval for videotaping. The participants also gave their assent to participate in the study.

The mean chronological age of the participants was 5.60 years (*SD* = 0.70; range 4.6–6.7) in the TD group and 5.51 years (*SD* = 0.44; range 4.7–6.3) in the ASD group, Mann-Whitney (*U*) = 127, *p* = 0.66. There was no significant difference between participants with ASD and those with typical development. Participants’ language developmental age was assessed by the language and communication subset in Psychoeducational Profile, Third Edition (PEP-3; Schopler et al., 2005). Trained experimenters gave instructions in Chinese (Cantonese), which followed the Chinese version of PEP-3 (Shek and Yu, 2014). The mean language developmental age of participants was 5.51 years (*SD* = 0.52; range 4.6–6.2) in the TD group and 5.38 years (*SD* = 0.38; range 4.6–6.2) in the ASD group, *U* = 118.5, *p* = 0.46. There was no significant difference in language developmental age between the two groups.

### Experimental Procedures

A narrative elicitation task was conducted by research assistants who were blind to the study design and hypotheses. The research assistants had been trained on the experimental procedures before the study. The instructions given to the research assistants were listed in Table 1. Six wordless pictures contained snapshots of a story about Tweety Bird and Sylvester were used. The story of Tweety Bird and Sylvester has been used in many prior studies to elicit speech and gestures. The story can be understood by both typical and atypical development across different cultures. Therefore, it is suitable for storytellers of different ages, different neurological conditions, and different language groups (McNeill, 1992, 2000). The story has a linear plot line about the two characters: Sylvester catches Tweety Bird, puts her in a sandwich, and tries to eat her.

**TABLE 1:** Instructions for experimenters in the narrative elicitation task.

Goal	Guideline/Example
1. Begin the story	“Let’s begin now.” “What’s happing?” “One day…”
2. Draw children’s attention	“Here is the next picture.”
3. Encourage the children	a. Repeat children’s speech “Yes, there is a bird.” b. Use open questions “What is next?” “What is the end of the story?” c. Praise the children “Your story is lovely. Can you tell me more?”

During the narrative elicitation task, the research assistant presented the pictures to each child in a temporal order. Firstly, the research assistant invited the child to generate a story form some pictures. Then the child was given two minutes to look at the pictures. When telling the story, the researcher was also allowed to interact with the child. In this way, the narrative elicitation approximated a natural setting. The researcher encouraged the child to produce a story that was as long and complete as possible. However, the researcher was not allowed to produce any words or gestures related to the pictures. To reduce the demand on memory recall, the research assistant kept presenting the pictures while the child was narrating. In this narrative elicitation task, the child needed to extract a coherent narrative from the pictures and represent it linguistically (Botting, 2002). By generating a story from several wordless pictures, we minimized the demand for language comprehension and recall of the materials (Demir et al., 2010). The task was videotaped for later transcription and analyses.

### Speech Transcription

Participants’ spoken narratives were transcribed by trained coders who were native Cantonese speakers and blind to the hypotheses of the research study. All words and pauses were transcribed and further segmented into separate utterances, with each utterance containing a character and its corresponding action [e.g., “The cat catches the bird (*zi2 maau1 sik6 zi2 zoek3 zai2*).”]. Clauses with more than one character or action were broken into two or more utterances [e.g., “The cat eats the bread but the bird escapes (*zi2 maau1 ngaau5 go3 min6 baau1 daan6 hai6 bei2 zi2 zoek3 zai2 zau2 lat1 zo2*)]” was coded as two utterances as “The cat eats the bread (*zi2 maau1 ngaau5 go3 min6 baau1*)” and “The bird escapes (*zi2 zoek3 zai2 zau2 lat1 zo2*)”. Utterances that did not contain information of the story were excluded from further analysis (e.g., “I have milk for breakfast.”). All transcriptions were checked by a second trained coder who was also a native Cantonese speaker and blind to the hypotheses.

### Gesture Coding

#### Identification of Gestures

All movements during narrations were coded by trained coders. The following movements were excluded: (1) hand movements that involved direct manipulation of an object (Goldin-Meadow et al., 1984); (2) motor stereotype and self-grooming movements (Silverman et al., 2017); (3) movements that did not contain information of the story (e.g., pointing to the fan on the wall).

#### Gesture Type

The present study followed a coding system initially described by McNeill (1992), who categorized co-speech gestures into four types: iconic, metaphoric, deictic, and beat. Iconic gestures resemble an aspect of the entity’s shape or movement (e.g., both hands flapping to represent a bird flying). Metaphoric gestures convey abstract ideas or concepts (e.g., thumb and index finger moving toward each other while saying “The bread is a little hard.”). Deictic or indexical gestures direct listeners’ attention to the specified entities by pointing at them with an index finger (e.g., pointing to the sandwich while saying “The bird is inside.”). Beat gestures are rhythmic hand movements that can segment and emphasize elements in speech (e.g., nodding while saying “Eat the bird.”). Additionally, emblems, which can refer to culture-specific meanings as single words (e.g., horizontal shake of the head means “no”) or phrases (e.g., shrugging the shoulders means “don’t know”) were also coded (de Marchena and Eigsti, 2010; Silverman et al., 2017).

#### Gesture Rate

Gesture rate was calculated as the number of gestures per utterance (total number of gestures divided by the total number of utterances).

#### Gesture Meaning and Gesture-Speech Relation

Each gesture was assigned a meaning based on its form and the co-occurring speech. The relationships between gesture meaning and co-occurring speech were categorized into three types depending on their semantic relationship (Özçalışkan and Goldin-Meadow, 2005; So et al., 2015; Wong and So, 2018). A reinforcing relation was coded when a gesture conveyed the same meaning as the co-occurring speech [e.g., shaking head when saying “The bird doesn’t want to go out (*zi2 zoek3 zai2 m4 soeng2 ceot1 heoi3*)”]. A supplementary relationship was coded when a gesture added extra information. That is, the meaning of the gesture was not explicitly conveyed in the co-occurring speech [e.g., saying “The cat wants to eat the bird (*zi2 maau1 soeng2 sik6 zo2 zi2 zoek3 zai2*)” and producing a CATCH gesture]. A disambiguating relation was coded when a gesture clarified an underspecified referent [e.g., saying “The cat went there (*zi2 maau1 heoi3 zo2 go2 dou6*)” and pointing to the right]. The number of each type of gesture-speech relation was counted.

#### Reliability

To assess the inter-coder reliability, 20% of the cases were randomly selected and independently coded by a second trained coder. The inter-coder agreement was 0.96 (*N* = 165, Cohen’s kappa = 0.96, *p* < 0.001) in an evaluation of gesture type and 0.86 (*N* = 165, Cohen’s kappa = 0.86, *p* < 0.001) in an evaluation of gesture-speech relation.

#### Statistical Analyses

The Mann-Whitney test was used to examine differences in gesture rate, gesture type, and gesture-speech relation between the two groups.

## Results

**FIGURE 1:**
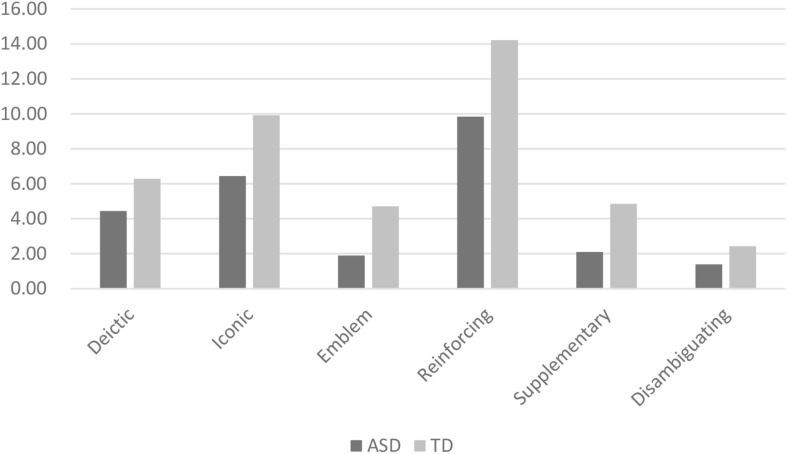
Number of gestures by gesture type and gesture-speech relation.

Table 1 shows the proportion of different gesture types and gesture-speech relation. Around one-third of the gestures produced during the storytelling task were deictic gestures, while iconic gestures accounted for about half of the total gestures in both groups. Most of the gestures (around 70%) represented a reinforcing meaning. Since the proportions of metaphoric gestures and beat gestures in gesture type were relatively small (less than 5%), they were excluded from the following analyses. Figure 1 shows the average number of gestures by gesture type and gesture-speech relation in the two groups.

As shown in Table 2, both groups produced similar numbers of utterances during storytelling. However, the children with ASD produced significantly fewer gestures, resulting in a lower gesture rate compared to the TD group. In addition, the children with ASD produced fewer emblems and supplementary gestures, while the numbers of deictic gestures, iconic gestures, reinforcing gestures, and disambiguating gestures they produced were comparable to the TD group (Table 3).

**TABLE 2:** Constitution of gestures produced by the ASD and TD group.

		Mean Proportion	
		ASD	TD
Gesture type	Deictic	33.3%	29.2%
	Iconic	48.3%	46.2%
	Metaphoric	1.1%	0%
	Beat	3.0%	2.7%
	Emblem	14.2%	21.9%
Gesture-speech relation	Reinforcing	73.8%	66.1%
	Supplementary	15.7%	22.6%
	Disambiguating	10.5%	11.3%
Deictic	Reinforcing	17.6%	17.3%
	Supplementary	5.2%	1.0%
	Disambiguating	10.5%	11.0%
Iconic	Reinforcing	38.6%	36.2%
	Supplementary	9.7%	10.0%
Emblem	Reinforcing	13.9%	10.6%
	Supplementary	0.4%	11.3%

**TABLE 3:** Participants’ characteristics, gestural skills, and comparison between the ASD and TD group.

	ASD (*n* = 20, 3 females)			TD (*n* = 14, 5 females)			Group comparison	
	Mean	SD	Range	Mean	SD	Range	*U*	*p*-value
Chorological age	5.51	0.44	4.7–6.3	5.60	0.70	4.6–6.7	127	0.66
Language developmental age	5.38	0.38	4.6–6.2	5.51	0.52	4.6–6.2	118.5	0.46
Utterances^a^	30.85	10.46	11–59	35.50	12.26	16–63	106.5	0.25
Gestures^b^	13.35	8.07	2–30	21.50	10.11	10–42	71	0.02*
Gesture rate^c^	0.44	0.23	0.1–1.0	0.61	0.22	0.4–1.1	80	0.004**
**Gesture type^d^**								
Deictic	4.45	3.99	0-13	6.29	5.62	0–21	111	0.32
Iconic	6.45	4.26	0-14	9.93	6.15	2–23	94.5	0.11
Emblem	1.90	2.22	0-8	4.71	4.78	0–18	83	0.05*
**Gesture-speech relation^e^**								
Reinforcing	9.85	6.56	0–22	14.21	7.23	6–29	96	0.13
Supplementary	2.10	2.15	0–7	4.86	4.26	0–17	73.5	0.02*
Disambiguating	1.40	1.85	0–6	2.43	2.77	0–9	100.5	0.15
**Deictic**								
Reinforcing	2.35	2.35	0–7	3.71	3.71	0–12		
Supplementary	0.70	0.92	0–3	0.21	0.85	0–2		
Disambiguating	1.40	1.85	0–6	2.36	2.65	0–9		
**Iconic**								
Reinforcing	5.15	3.59	0–10	7.79	4.85	2–17		
Supplementary	1.30	1.75	0–5	2.14	1.99	0–6		
**Emblem**								
Reinforcing	1.85	2.23	0–8	2.29	2.09	0–7		
Supplementary	0.05	0.22	0–1	2.43	3.84	0–14		

^*a*^Total number of utterances. ^*b*^Total number of gestures. ^*c*^Total number of gestures divided by the total number of utterances. ^*d*^Number of each gesture type. ^*e*^Number of each gesture-speech relation. *Significant at 0.05 level; **significant at 0.01 level.

We further analyzed the constitution of emblems by gesture-speech relation. Results showed that children with ASD tended to use emblems to reinforce accompanying speech (97.4%), while TD children did not show this tendency (51.5%). Besides, we analyzed the constitution of supplementary gestures by gesture type (deictic, iconic, and emblem). We found that in the TD group, half (50%) of the supplementary gestures were emblems, followed by 44.1% of iconic gestures. Deictic gestures only made up less than 5% (4.4%) of the supplementary gestures. In sharp contrast, only 2.4% of the supplementary gestures were emblems in the ASD group. Around two-thirds (61.9%) were iconic gestures and one-third (33.3%) were deictic gestures.

## Discussion

Results of the present study showed that the children with ASD had a lower gesture rate, which is consistent with the findings reported by So et al. (2015) and Silverman et al. (2017), whose participants were either school-age children or adolescents. In addition, echoing the findings of So et al. (2015) for school-age children, we found that the children with ASD produced fewer emblems than their TD peers, indicating that a delay in producing emblems exists in early and middle childhood. The children with ASD also had a delay in producing supplementary gestures, which was also reported by Morett et al. (2016) and So et al. (2015) in regard to autistic participants attending primary or middle school. These findings suggest that the impairment of gestural skills in individuals with ASD appears from preschool age and persists when they grow up.

By analyzing the constitution of emblems by gesture-speech relation, we found that most of the emblems produced by ASD had their meaning conveyed in the co-occurring speech, while TD produced half of the emblems without saying their meanings. Emblems, also known as conventional gestures, have culture-specific meanings and forms. However, children with ASD may not realize that emblems could be produced and understood in a supplementary way. One possible explanation is that while children with ASD could learn some gestural skills from daily life as their TD peers (Wise and Sevcik, 2012), they are more likely to learn the gestures that are produced in a reinforcing way, in which the connection between the gesture and its corresponding meaning is explicit and clear (Knutsen et al., 2017). Therefore, they may have difficulty in learning emblems, which are more likely to be produced to supplement speech in daily life compared to other types of gestures (McNeill, 1992). Besides, children with ASD may be more likely to learn emblems that reinforce co-occurring speech, and produce them in the same way: reinforcing, rather than supplementary.

The delay in producing emblems may be a possible cause of impairment in producing supplementary gestures. Compared to other types of gestures, emblems can be used and understood without accompanying speech. These findings showed that compared to other types of gestures, TD children tended to produce emblems in a supplementary way, which is consistent with previous studies (McNeill, 1992, 2000). So et al. (2015) further pointed out that impairment in producing supplementary gestures may be due to the inability of individuals with ASD to integrate cross-modal semantic information. To produce gestures to supplement co-occurring speech, children have to coordinate information from both verbal language and hand movement, which may be more difficult for individuals with ASD than those with typical development. Notably, around one-third of supplementary gestures were deictic gestures in ASD. Producing deictic gestures to supplement speech (e.g., saying “eat” when pointing to the bread) is regarding as an early stage of development in both verbal language and gestures (Iverson and Goldin-Meadow, 2005; Özçalışkan et al., 2016). When children manage single words, they begin to use a gesture-plus-word combination (e.g., a verb + pointing) as two-word phrases, which is usually observed around 18 to 24 months in TD children (Iverson and Goldin-Meadow, 2005). Using deictic gestures in a supplementary way indicated that there may be some delay in gestural development in ASD.

However, unlike Camaioni et al. (2003) and Luyster et al. (2007), we did not find impairments in producing deictic and iconic gestures in the ASD group. Besides, de Marchena and Eigsti (2010) and de Marchena et al. (2019) reported no difference or marginally significant difference in gesture rate, which are not consistent with the results in this study. There are three possible reasons for these contradictory findings. One is about the task design. Some researchers have proposed that task design differences may result in variations across studies in language development, including narrative productions and gestural skills (Berman, 2004; Stirling et al., 2014). For example, de Marchena et al. (2019) found that participants in the ASD group used some types of gestures more often than those in the TD group in a collaborative referential communication task. These gestures were used to regulate turn-taking, which is not included in a storytelling task. Therefore, similar results may not be observed in the present study. Besides, some tasks may correlate with other social cognitive abilities. For example, asking children to retell a story to a stranger who had never read the story requires children’s theory of mind understanding (Stirling et al., 2014). The last possible reason is age, Participants in de Marchena and Eigsti (2010) were adolescents and those in de Marchena et al. (2019) were adults, who may use gestures differently from preschool children. Therefore, it is critical to administer different tasks, as well as combine different findings, to obtain a better understanding of gestural skills in individuals with ASD. The second possible reason is the difference in the calculation of gesture rate. For example, de Marchena et al. (2019) defined gesture rate as the number of gestures per minute, while this study defined it as the number of gestures per utterance. In addition, the difference in the categories of gesture type is common. Apart from the gesture types used in this study, some researchers use categories including descriptive gestures, symbolic gestures, interactive gestures, and numerical gestures (Ingersoll, 2007; de Marchena et al., 2019). These differences in definition and characterization make it difficult to compare results across studies and to reach an agreement. Besides, the small sample size and inequality in sex ratio between the two groups may affect the results. Although we were not able to draw conclusions on the gestural impairment in ASD from this study, our findings show the differences in gestural use in TD and ASD. These findings could provide evidence for gestural training programs for children with ASD at an early age.

## Data Availability Statement

The raw data supporting the conclusions of this article will be made available by the authors, without undue reservation.

## Ethics Statement

The studies involving human participants were reviewed and approved by Institutional Review Board, the Chinese University of Hong Kong (Reference no. 14600817). Written informed consent to participate in this study was provided by the participants’ legal guardian/next of kin.

## Author Contributions

YH: conceptualization, data curation, formal analysis, investigation, and writing – original draft. MW: data curation, project administration, and validation. W-YL: data curation. C-HC: data curation and validation. W-CS: conceptualization, methodology, funding acquisition, resources, supervision, and writing – review and editing. All authors contributed to the article and approved the submitted version.

## Conflict of Interest

The authors declare that the research was conducted in the absence of any commercial or financial relationships that could be construed as a potential conflict of interest.
